# External validation of a commercial AI system for pulmonary embolism detection on chest CTPA: a multicenter study

**DOI:** 10.3389/fmolb.2026.1774152

**Published:** 2026-03-11

**Authors:** Mireayi Tudi, Saimaitikari Abudoubari, Xierenayi Waresi, Aikebaierjiang Ainiwaer, Nuermaimaijiang Abudouwufu, Palidanmu Wumaier, Adilijiang Abula, Yuwei Xia, Ailiyaerjiang Aisika, Ya Qiu, Maimaitiaili Tuerxun, Abudouresuli Tuersun

**Affiliations:** 1 Department of Infection, The First People’s Hospital of Kashi (Kashgar) Prefecture, Kashi, China; 2 Department of Radiology, The First People’s Hospital of Kashi (Kashgar) Prefecture, Kashi, China; 3 The Xinjiang Key Laboratory of Artificial Intelligence Assisted Imaging Diagnosis, Kashi, China; 4 Department of Geriatrics, Shache County People’s Hospital, Kashi, China; 5 School of Mathematics and Statistics, Kashi University, Kashi, China; 6 Shanghai United Imaging Intelligence Medical Technology Co., Ltd., Shanghai, China; 7 Department of Radiology, The Fourth Affiliated Hospital of Xinjiang Medical University (Xinjiang Hospital of Traditional Chinese Medicine), Urumqi, Xinjiang, China

**Keywords:** artificial intelligence, computed tomography, CT pulmonary angiography, pulmonary artery obstruction index, pulmonary embolism

## Abstract

**Background:**

Pulmonary embolism (PE) is a critical cardiovascular emergency requiring prompt, accurate diagnosis. CT pulmonary angiography (CTPA) is the diagnostic gold standard, yet rising case volumes and radiologist shortages challenge clinical workflows. Artificial intelligence (AI) offers potential to enhance diagnostic precision and efficiency. This multicenter study validates the performance of a commercially available AI system compared with radiologist interpretation alone and in combination.

**Methods:**

In this retrospective analysis, 600 consecutive patients suspected of PE underwent CTPA between January 2024 and May 2025 at three hospitals in Xinjiang. All scans employed 256-slice CT with standardized protocols (100 kV, 0.625 mm slice thickness, iohexol contrast). Images were processed using uAIDiscover PE software, generating Pulmonary Thrombus Burden Score (PTBS). Manual Pulmonary Artery Obstruction Index (PAOI) was independently scored via the Qanadli system by consensus of three senior radiologists, serving as the reference standard. Diagnostic accuracy and correlation between AI and manual scores were assessed (SPSS 24.0; P < 0.05).

**Results:**

Among 600 patients analyzed, 271 (45.2%) had pulmonary embolism. PE patients had significantly higher BMI and greater prevalence of hypertension and coronary artery disease (P < 0.05). ROC analysis demonstrated superior diagnostic performance for the combined manual + AI approach across all centers (AUC: 0.928–0.934) compared to AI alone (AUC: 0.807–0.810) or manual reading alone (AUC: 0.888–0.914). AI processing was remarkably fast at 0.19 ± 0.02 min versus 5.26 ± 0.94 min for radiologists alone, while combined approach required 2.61 ± 0.69 min. Strong correlation was observed between AI-derived PTBS and manually calculated PAOI (r = 0.863, P < 0.001). The combined approach significantly reduced diagnostic errors to 7 cases compared to 43 for AI alone and 29 for manual reading alone.

**Conclusion:**

Integration of AI with manual interpretation improves pulmonary embolism detection accuracy and reduces reading time, supporting its implementation to optimize clinical workflow and patient outcomes.

## 1 Introduction

Pulmonary embolism (PE) is a common and potentially life-threatening manifestation of venous thromboembolism that imposes a substantial global burden on patients and health systems (Marshall et al., 2011; Albeladi et al., 2025). It remains one of the leading acute cardiovascular emergencies requiring rapid clinical assessment (Miniati et al., 2012). Clinical presentations of PE vary widely, ranging from incidental, small subsegmental emboli to massive, hemodynamically unstable events. Even survivors of the acute phase are at risk of substantial morbidity, including recurrent venous thromboembolism and chronic thromboembolic pulmonary hypertension, which often leads to persistent functional impairment (Bĕlohlávek et al., 2013; Shin Lee et al., 2025). Timely and accurate detection is critical: prompt anticoagulation or reperfusion therapy in appropriately risk-stratified patients reduces mortality and major complications, whereas diagnostic delays or missed diagnoses result in preventable deterioration, which underscores the need for reliable, rapid diagnostic pathways for PE in routine clinical practice (Giordano et al., 2017; Zhong et al., 2025).

CT pulmonary angiography (CTPA) is the gold standard imaging modality for PE detection, diagnostic confirmation, and evaluation of clot burden and right ventricular strain (Xi et al., 2025; Uchiyama et al., 2025). Despite its central role, several pragmatic and technical limitations undermine diagnostic performance and throughput (Meng et al., 2025; Duan et al., 2025). Globally increasing case volumes, coupled with a shortage of experienced thoracic radiologists, prolong interpretation times and may delay treatment (Jin et al., 2025; Wu et al., 2025). Image-level issues, such as hard-to-see subsegmental emboli, inadequate or mistimed contrast, motion, and artifacts, reduce sensitivity, and interobserver variability in detecting and quantifying embolic burden further exacerbates these problems and limits reproducibility across centers (Sauter et al., 2025; Taguchi et al., 2025). Therefore, developing accurate, fast diagnostic tool is necessary.

Recent advances in deep learning have accelerated the application of AI to CTPA interpretation for PE, moving beyond research prototypes to clinically deployed tools that assist with detection, triage, and automated segmentation, quantification of embolic burden (Abed et al., 2025; Wysokinski et al., 2024). Convolutional neural networks and transformer-based models optimize feature extraction across volumetric CT data to flag central and increasingly subtle subsegmental emboli, prioritize studies for rapid review, and generate consistent clot maps and right-ventricular/left-ventricular ratio estimates that support risk stratification (Lanza et al., 2025; Shen et al., 2025). Several commercial vendors now integrate into picture archiving and communication system (PACS) workflows, enabling near-real-time notification to radiologists and emergency teams.

Nonetheless, the real-world clinical performance of AI-based PE diagnostic software requires rigorous external validation to assess limitations and ensure reliability. This study, conducted within routine clinical workflows, aims to systematically compare the embolus detection accuracy and diagnostic performance of a commercial AI-assisted system against radiologist interpretation, alongside evaluation of associated time efficiency metrics, to establish its practical clinical utility.

## 2 Methods

### 2.1 Study population

This multicenter retrospective study included 600 patients with suspected pulmonary embolism (PE) who underwent CT pulmonary angiography (CTPA) between January 2024 and May 2025. Patients were recruited from three institutions: Kashgar First People’s Hospital (n = 360), the Fourth Affiliated Hospital of Xinjiang Medical University (n = 120), and Shache County People’s Hospital (n = 150).

**FIGURE 1:**
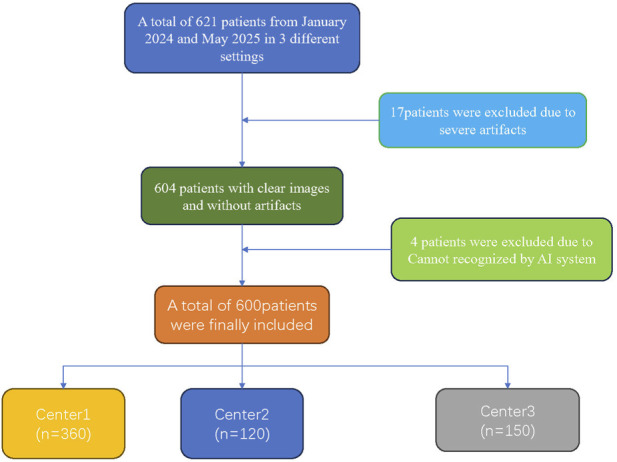
Inclusion and exclusion criteria.

Inclusion criteria comprised high-risk patients for PE who underwent CTPA. Exclusion criteria were: (1) cases that could not be uploaded to or processed by the AI system; (2) examinations with severe imaging artifacts rendering them non-diagnostic; and (3) reports that were equivocal and could not be confirmed. After applying these criteria, 600 patients were ultimately enrolled. The patient selection flowchart is presented in Figure 1.

### 2.2 Ethical approval

Ethical approval for this retrospective study was obtained from the Institutional Review Boards of The First People’s Hospital of Kashi Prefecture, the Fourth Affiliated Hospital of Xinjiang Medical University, and Shache County People’s Hospital. Informed consent was waived due to the study’s retrospective design.

### 2.3 Data acquisition

At Centers 1 and 2, examinations were performed on a 256-slice CT scanner (Revolution CT, GE Healthcare), while at Center 3, a 128-slice CT scanner (Siemens Healthcare) was used. All patients were positioned supine and headfirst. Scan coverage extended from the thoracic inlet to the costophrenic angles.

Following a localizer scan, CTPA was performed using the following parameters: tube voltage 100 kV; automatic tube current modulation; rotation time 1.0 s/rotation; pitch 0.984; reconstructed slice thickness 0.625 mm. Bolus-tracking technique was employed with the region of interest placed in the main pulmonary artery, a triggering threshold of 100 Hounsfield units, and a 3-s delay before automatic scan initiation. Iohexol (320 mg I/mL) was administered via the right median cubital vein at 4–5 mL/s (total volume 30–40 mL), followed by a 30 mL saline flush at the same rate.

Acquired images were saved in DICOM format and automatically uploaded via the institutional network to the picture archiving and communication system (PACS) and to uAI DiscoverPE (United Imaging Intelligence, Shanghai, China), an AI-assisted pulmonary embolism screening and diagnostic software, for automated analysis.

### 2.4 Reference standard and case adjudication

The reference standard for pulmonary embolism diagnosis was established through consensus review. Initial diagnostic reports were issued by senior radiologists with more than 10 years of clinical experience. Each case was then independently reviewed by two additional radiologists with more than 15 and 20 years of experience, respectively. Discrepant interpretations between radiologists or between AI and radiologists were resolved via consensus discussion involving all three senior radiologists to establish the final diagnosis. Cases deemed equivocal—where consensus diagnosis of pulmonary embolism presence or absence could not be confidently established—were excluded from analysis to prevent uncertainty-related bias and maintain a robust diagnostic reference standard. This approach ensures that reported diagnostic metrics focus on well-defined positive and negative cases for PE.

### 2.5 Image interpretation

#### 2.5.1 Radiologist interpretation

At each of the three centers, two radiologists with more than 5 years of experience independently reviewed all CTPA examinations in a blinded manner, without access to AI-generated results or clinical information beyond the imaging indication. Diagnostic time for each radiologist interpretation was recorded.

#### 2.5.2 AI-assisted interpretation

The AI system (uAI DiscoverPE) automatically analyzed all uploaded CTPA studies and generated: (1) a binary classification (PE present or absent); (2) automated segmentation and localization of detected emboli; and (3) a quantitative Pulmonary Thrombus Burden Score (PTBS). Processing time for AI analysis was recorded for each case (detailed algorithm of uAI DiscoverPE are provided in Supplementary Material Algorithm Implementation of the uAI DiscoverPE System).

#### 2.5.3 Combined radiologist + AI interpretation

Following independent radiologist and AI assessments, a combined interpretation was performed in which radiologists reviewed cases with access to AI-generated results, including embolus localization maps and PTBS. Diagnostic time for this combined approach was recorded.

#### 2.5.4 Blinding and reading procedures

Radiologists performed initial manual readings blinded to AI outputs and clinical information to avoid interpretation bias. The AI system independently analyzed all CTPA images. Subsequently, combined reading involved radiologists reviewing AI-flagged findings after completing the initial independent interpretations, reflecting a practical support workflow. This sequential process was designed to minimize potential bias introduced by knowledge of AI results during primary interpretations.

### 2.6 Pulmonary artery obstruction index and thrombus burden assessment

#### 2.6.1 Manual PAOI calculation

The Pulmonary Artery Obstruction Index (PAOI) was manually calculated using the Qanadli scoring system (Soffer et al., 2021), a validated CTPA-based method for quantifying clot burden. The pulmonary arterial tree was divided into 20 segmental arteries (10 per lung), each assigned a location weight (n). The degree of obstruction (d) at each affected segment was scored as: 1 point for partial obstruction (luminal narrowing without complete occlusion) and 2 points for complete obstruction (total occlusion with no distal contrast opacification). The PAOI was calculated as:

$$\text{PAOI}=\sum \left(n\times d\right)$$

#### 2.6.2 AI-generated thrombus burden score

The AI system automatically generated a Pulmonary Thrombus Burden Score (PTBS) for each case, designed to quantify the anatomic extent of thrombotic occlusion within the pulmonary vasculature. Given the conceptual similarity between PTBS and PAOI, correlation analysis was performed to assess agreement between these metrics.

### 2.7 Statistical analyses

Statistical analyses were performed using SPSS version 24.0 (IBM Corp., Armonk, NY, United States). Categorical variables are presented as counts and percentages, and continuous variables as mean ± standard deviation. For the manual (human), AI, and combined diagnostic approaches, accuracy, sensitivity, specificity, positive predictive value (PPV), negative predictive value (NPV), and receiver operating characteristic (ROC) curve area under the curve (AUC) were calculated. Differences between AUCs were compared using the DeLong test, and differences in diagnostic time between groups were assessed with a nonparametric rank-sum test. Interrater reliability of the reference standard was evaluated to assess the reproducibility of the consensus diagnosis by three radiologists. Cohen’s Kappa coefficient was used for pairwise interrater agreement, and Fleiss’ Kappa coefficient was adopted to determine the overall interrater agreement among the three radiologists. A two-sided P value <0.05 was considered statistically significant for all analyses.

## 3 Results

### 3.1 Baseline character

Baseline characteristics are summarized in Table 1. A total of 600 patients were included, of whom 271 had pulmonary embolism (PE) and 329 did not (non-PE). The median age was similar between groups (non-PE: 53 [P25, P75 59, 64] years vs. PE: 55 [59, 65] years; U = 1.686, P = 0.092). Overall, 254 patients (42.3%) were male (non-PE 138/329, 41.9%; PE 116/271, 42.8%) and 346 (57.7%) were female, with no significant sex difference between groups (χ^2^ = 0.045, P = 0.832). Current smoking was present in 319 patients (53.2%) and alcohol use in 305 (50.8%), with no between-group differences (smoking: non-PE 177/329 [53.8%] vs. PE 142/271 [52.4%], χ^2^ = 0.117, P = 0.732; alcohol: non-PE 177/329 [53.8%] vs. PE 128/271 [47.2%], χ^2^ = 2.564, P = 0.109). Body mass index differed significantly: median BMI was higher in the PE group (non-PE: 22.25 [P25, P75 23.2, 24.1] vs. PE: 23.4 [25.3, 27.2]; U = 11.767, P < 0.001). Hypertension and coronary artery disease (CHD) were more frequent among patients with PE (hypertension: PE 150/271 [55.4%] vs. non-PE 148/329 [45.0%], χ^2^ = 6.387, P = 0.011; CHD: PE 157/271 [57.9%] vs. non-PE 150/329 [45.6%], χ^2^ = 9.057, P = 0.003). Distribution across the three study centers did not differ significantly (χ^2^ = 2.269, P = 0.322).

**TABLE 1:** Baseline information.

Variable	nonPE (n = 329) N (%), M(P_25_, P_75_)	PE (n = 271) N (%), M(P_25_, P_75_)	All (n = 600) N (%), M(P_25_, P_75_)	统计量 (X^2^, U)	P
Gender					
Male	138 (41.9)	116 (42.8)	254 (42.3)	0.045	0.832
Female	191 (58.1)	155 (57.2)	346 (57.7)		
Age	53 (59, 64)	55 (59, 65)	54 (59, 65)	1.686	0.092
BMI	22.25 (23.2, 24.1)	23.4 (25.3, 27.2)	22.6 (23.7, 25.2)	11.767	<0.001
Smoking					
Non	152 (46.2)	129 (47.6)	281 (46.8)	0.117	0.732
Yes	177 (53.8)	142 (52.4)	319 (53.2)		
Alcohol					
No	152 (46.2)	143 (52.8)	295 (49.2)	2.564	0.109
Yes	177 (53.8)	128 (47.2)	305 (50.8)		
HBP					
No	181 (55)	121 (44.6)	302 (50.3)	6.387	0.011
Yes	148 (45)	150 (55.4)	298 (49.7)		
CHD					
No	179 (54.4)	114 (42.1)	293 (48.8)	9.057	0.003
Yes	150 (45.6)	157 (57.9)	307 (51.2)		
Center					
1	195 (59.3)	150 (55.4)	345 (57.5)	2.269	0.322
2	65 (19.8)	50 (18.5)	115 (19.2)		
3	69 (21)	71 (26.2)	140 (23.3)		

HBP, high blood pressure; CHD, coronary artery disease.

### 3.2 Interrater reliability of the reference standard

As shown in Table 2, excellent pairwise interrater agreement was observed between each two radiologists, with Cohen’s Kappa values ranging from 0.867 to 0.894 (all P < 0.001). The overall interrater agreement among the three radiologists was also excellent, with a Fleiss’ Kappa value of 0.884 (P < 0.001). These results confirmed high reproducibility and reliability of the reference standard established by consensus of the three senior radiologists, which further strengthened the credibility of the subsequent diagnostic performance comparison among different approaches.

**TABLE 2:** Interrater agreement for pulmonary embolism diagnosis among three senior radiologists.

Comparison groups		Kappa value	P
Radiologist 1	Radiologist 2	0.867	<0.001
Radiologist 1	Radiologist 3	0.879	<0.001
Radiologist 2	Radiologist 3	0.894	<0.001
All three radiologists (Fleiss’ Kappa)		0.884	<0.001

### 3.3 Diagnostic performance of radiologist-only, AI-only, and combined approaches

Diagnostic performance metrics for all three approaches are presented in Table 3. Receiver operating characteristic (ROC) analysis demonstrated that the combined radiologist + AI approach yielded the highest discriminative performance for pulmonary embolism detection across all centers.

**TABLE 3:** Comparison of diagnostic efficacy of AI, radiologists, and AI combined with radiologists in 3 centers.

Centers	Method	AUC (95% CI)	Sen (95% CI)	Spe (95% CI)	PPV (95% CI)	NPV (95% CI)
Center 1	AI	0.8085 (0.7673–0.8466)	0.8067 (0.7361–0.8619)	0.8103 (0.7494–0.8591)	0.7658 (0.6940–0.8251)	0.8449 (0.7862–0.8898)
	Manual	0.8879 (0.8553–0.9181)	0.8733 (0.8106–0.9174)	0.9026 (0.8528–0.9367)	0.8733 (0.8106–0.9174)	0.9026 (0.8528–0.9367)
	AI + manual	0.9305 (0.9017–0.9546)	0.9533 (0.9068–0.9772)	0.9077 (0.8588–0.9408)	0.8882 (0.8302–0.9281)	0.9620 (0.9236–0.9815)
	AI	0.8100 (0.7391–0.8847)	0.8200 (0.6920–0.9023)	0.8000 (0.6873–0.8792)	0.7593 (0.6305–0.8536)	0.8525 (0.7428–0.9204)
Center 2	Manual	0.9038 (0.8483–0.9569)	0.9000 (0.7864–0.9565)	0.9077 (0.8129–0.9570)	0.8824 (0.7662–0.9449)	0.9219 (0.8298–0.9662)
	AI + manual	0.9338 (0.8854–0.9744)	0.9600 (0.8654–0.9890)	0.9077 (0.8129–0.9570)	0.8889 (0.7781–0.9481)	0.9672 (0.8881–0.9910)
	AI	0.8072 (0.7349–0.8717)	0.8028 (0.6958–0.8787)	0.8116 (0.7039–0.8865)	0.8143 (0.7077–0.8881)	0.8000 (0.6918–0.8770)
Center 3	Manual	0.9143 (0.8648–0.9571)	0.9155 (0.8276–0.9607)	0.9130 (0.8230–0.9595)	0.9155 (0.8276–0.9607)	0.9130 (0.8230–0.9595)
	AI + manual	0.9281 (0.8815–0.9707)	0.9577 (0.8830–0.9855)	0.8986 (0.8051–0.9500)	0.9067 (0.8197–0.9541)	0.9538 (0.8729–0.9842)

Detailed pairwise AUC comparisons among the three diagnostic approaches for each center and the overall cohort are shown in Table 4. The combined AI + Manual approach had significantly higher AUC than AI-only in all centers and the overall cohort (all P < 0.05); the overall cohort also showed a significant AUC difference between Manual and AI + Manual approaches (P = 0.0427).

**TABLE 4:** Pairwise comparisons of AUC among three diagnostic approaches (center-specific and overall cohort).

Centers	Comparison group	Z	P
Center 1	AI vs. manual	−2.837	0.004
	AI vs. AI + manual	−4.771	<0.001
	Manual vs. AI + manual	−1.890	0.059
Center 2	AI vs. manual	−1.892	0.058
	AI vs. AI + manual	−2.824	0.004
	Manual vs. AI + manual	−0.803	0.422
Center 3	AI vs. manual	−2.4162	0.0157
	AI vs. AI + manual	−2.7335	0.0063
	Manual vs. AI + manual	−0.4084	0.6830
Overall	AI vs. manual	−4.0184	<0.001
	AI vs. AI + manual	−6.0111	<0.001
	Manual vs. AI + manual	−2.0264	0.0427

**FIGURE 2:**
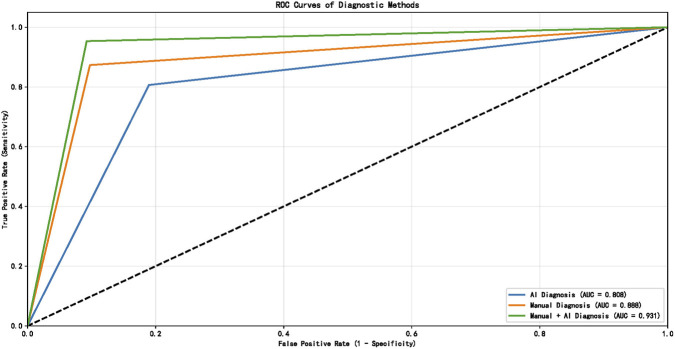
ROC curves in Center 1.

**FIGURE 3:**
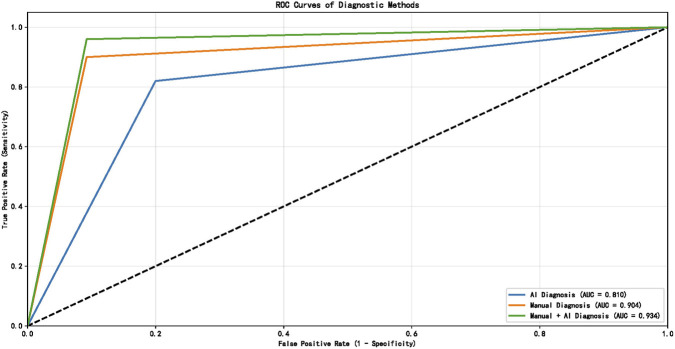
ROC curve in Center 2.

**FIGURE 4:**
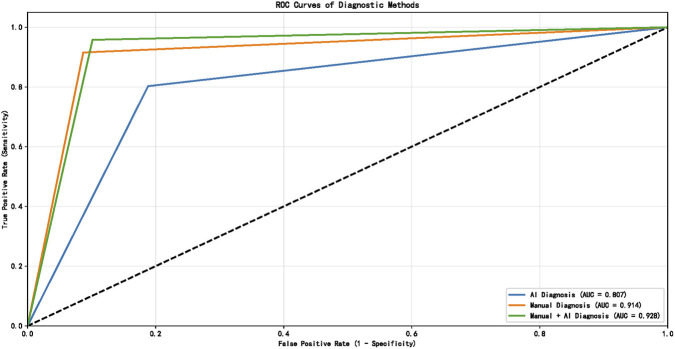
ROC curve in Center 3.

In Center 1, AUC values were: AI-only = 0.808, radiologist-only = 0.888, combined = 0.931 (Figure 2). In Center 2: AI-only = 0.810, radiologist-only = 0.904, combined = 0.934 (Figure 3). In Center 3: AI-only = 0.807, radiologist-only = 0.914, combined = 0.928 (Figure 4).

DeLong test comparisons revealed statistically significant improvements in AUC for the combined approach compared to either AI-only or radiologist-only interpretation across all centers (P < 0.05 for all comparisons).

### 3.4 Diagnostic speed comparison

Diagnostic speed was evaluated by comparing interpretation times across the three diagnostic approaches in all participating centers (Table 5). The AI system demonstrated consistently rapid processing times, requiring only 0.19 ± 0.02 min (approximately 11.4 s) across all centers. In contrast, radiologist interpretation alone required significantly longer processing times, averaging 5.26 ± 0.94 min across all centers. The combined AI-assisted radiologist approach resulted in intermediate interpretation times of 2.61 ± 0.69 min overall, representing a substantial reduction compared to radiologist-only interpretation.

**TABLE 5:** Comparison of diagnostic speed of AI, radiologists, and AI combined with radiologists in 3 centers.

Centers	AI	Manual	Manual & AI	AI vs. Manual		AI vs. combine		Manual vs. combine	
				*Z*	*P*	*Z*	*P*	*Z*	*P*
Center 1	0.19 + 0.02	5.26 + 0.94	2.61 + 0.69	−22.764	<0.001	−22.761	<0.001	−22.172	<0.001
Center 2	0.19 + 0.03	5.28 + 0.96	2.62 + 0.70	−13.125	<0.001	−13.123	<0.001	−12.740	<0.001
Center 3	0.20 + 0.03	5.24 + 0.95	2.63 + 0.70	−14.486	<0.001	−14.484	<0.001	−14.077	<0.001
All	0.19 + 0.03	5.26 + 0.94	2.61 + 0.69	−30.030	<0.001	−30.025	<0.001	−29.213	<0.001

### 3.5 Correlation between AI-generated and manual thrombus burden scores

The correlation between AI-derived PTBS and manually calculated PAOI was evaluated across all participating centers (Table 6). The PTBS was automatically generated by the AI system as a quantitative measure of pulmonary embolic burden, while the PAOI was manually calculated by experienced radiologists using the standardized Qanadli scoring method.

**TABLE 6:** Correlation analysis of PAOI s and PTBS.

Index	PTBS Center 1 (%)		PTBS Center 2 (%)		PTBS Center 3 (%)		PTBS All (%)	
	r	*P*	r	*P*	r	*P*	r	*P*
PAOI Center 1 (%)	0.866	<0.001						
PAOI Center 1 (%)			0.824	<0.001				
PAOI Center 1 (%)					0.909	<0.001		
PAOI All (%)							0.863	<0.001

Strong positive correlations were consistently observed between PTBS and PAOI across all centers. Center 1 demonstrated an excellent correlation coefficient of r = 0.866 (P < 0.001), while Center 2 showed similarly robust correlation with r = 0.824 (P < 0.001). Center 3 achieved the highest correlation coefficient at r = 0.909 (P < 0.001). When analyzing the combined dataset from all centers, the overall correlation remained strong at r = 0.863 (P < 0.001).

### 3.6 Misdiagnosed cases

**FIGURE 5:**
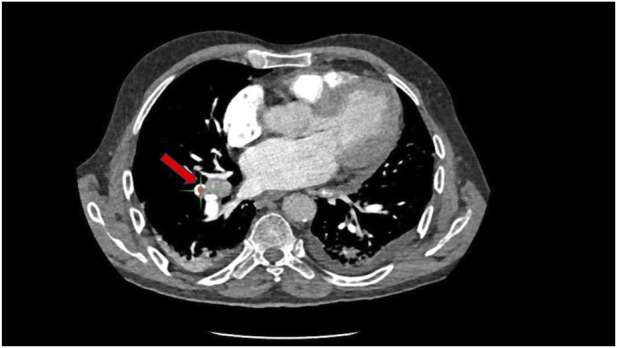
Misclassified case by AI system (perihilar lymph nodes mistaken for pulmonary embolism).

Compared with the reference standard across the three centers, the AI algorithm produced 43 diagnostic errors (7 false-negative and 36 false-positive cases; representative examples shown in Figure 5). Radiologist-only interpretation yielded 29 diagnostic errors (17 false-negative and 12 false-positive cases). The combined radiologist + AI approach resulted in only 7 diagnostic errors (2 false-negative and 5 false-positive cases), representing a 75.9% reduction in errors compared to AI alone and a 75.9% reduction compared to radiologists alone.

## 4 Discussions

The diagnostic accuracy of radiologists in detecting pulmonary embolism (PE) on computed tomography pulmonary angiography (CTPA) has been extensively documented in the literature, with reported sensitivities ranging from 0.67 to 0.87 and specificities from 0.89 to 0.99 (Soffer et al., 2021; Rothenberg et al., 2023; Kligerman et al., 2018). While these performance metrics demonstrate considerable expertise among radiological professionals, they also highlight the inherent challenges in PE detection and the potential for diagnostic errors that can have significant clinical consequences (Hutchinson et al., 2015). The complexity of PE diagnosis is further compounded by factors such as reader experience, image quality, patient body habitus, and the anatomical location of emboli. In recognition of these challenges and the critical importance of timely PE diagnosis, the United States Food and Drug Administration (FDA) has increasingly prioritized the development and validation of artificial intelligence algorithms specifically designed for PE detection on CTPA imaging (Rothenberg et al., 2023). This regulatory emphasis reflects a broader initiative to optimize rapid screening pathways for patients with clinically.

Recent advances in machine learning and deep learning technologies have demonstrated remarkable capabilities in medical image analysis, particularly in the detection and classification of pulmonary emboli on CTPA studies (Weikert et al., 2020; Vallée et al., 2024). Contemporary studies have shown that sophisticated AI algorithms can identify and characterize PE with exceptional speed, often completing comprehensive analyses within seconds to minutes compared to the traditional radiological interpretation time (Langius-Wiffen et al., 2024). These technological developments have culminated in several AI-powered solutions receiving FDA clearance for clinical implementation, specifically designed for computer-aided detection (CAD) and automated triage functions. These cleared systems represent a significant milestone in the integration of artificial intelligence into routine clinical practice, offering the potential to streamline patient management workflows, reduce interpretation variability, and enhance overall diagnostic confidence (Lanza et al., 2025). However, it is important to acknowledge that AI systems are not static entities; they require continuous training, validation, and iterative refinement to maintain and enhance their diagnostic accuracy and clinical utility. This ongoing development process necessitates sustained collaboration between technology developers, radiologists, and clinical researchers to ensure optimal performance across diverse patient populations and clinical scenarios.

The findings of our comprehensive analysis reveal a persistent performance gap between standalone AI systems and experienced radiologists in PE detection. Our results demonstrate that the AUC for the combined radiologist and AI reading approach (92.81%–93.38%) was substantially superior to both radiologists working independently (88.79%–91.43%) and AI systems operating in isolation (80.72%–81%). These findings are consistent with and corroborate the results reported by Soffer et al. (2021), who similarly demonstrated the synergistic benefits of human-AI collaboration in radiological diagnosis. The superior performance of the combined approach suggests that AI systems function most effectively as assistive tools rather than replacement technologies, augmenting human expertise rather than superseding it. This collaborative model has the potential to significantly reduce radiologists’ cognitive workload, particularly in high-volume clinical environments where rapid and accurate diagnosis is paramount.

A critical advantage of AI implementation in clinical practice is the substantial reduction in diagnostic interpretation time, which directly impacts patient care efficiency and workflow optimization. Our analysis demonstrated that AI systems achieved remarkably rapid processing times of 0.19 ± 0.02 min (approximately 11.4 s) across all participating centers, representing a dramatic improvement over traditional radiologist interpretation times of 5.26 ± 0.94 min. The combined AI-assisted approach yielded intermediate processing times of 2.61 ± 0.69 min, achieving a 50% reduction in interpretation time compared to manual reading alone while maintaining superior diagnostic accuracy. This temporal efficiency gain is particularly significant in emergency department settings and high-volume imaging centers, where rapid turnaround times are essential for optimal patient management. The ability to provide near-instantaneous preliminary assessments through AI triage systems can facilitate earlier clinical decision-making, potentially reducing time to anticoagulation initiation in confirmed PE cases and expediting appropriate discharge pathways for patients with negative studies.

The strong correlation observed between AI-generated Pulmonary Thrombus Burden Score (PTBS) and manually calculated Pulmonary Artery Obstruction Index (PAOI) across all centers (r = 0.863, P < 0.001) validates the reliability of automated quantitative assessment tools in clinical practice. This correlation analysis, with individual center coefficients ranging from r = 0.824 to r = 0.909, demonstrates consistent performance across different institutional settings and radiologist expertise levels. The automated generation of standardized thrombus burden metrics represents a significant advancement in PE risk stratification, as manual PAOI calculations are time-intensive and subject to inter-observer variability. The ability of AI systems to provide rapid, objective, and reproducible quantitative assessments of embolic burden has important implications for treatment planning, as higher clot burdens are associated with increased risk of hemodynamic compromise and mortality. This automated quantification capability enables more consistent risk stratification protocols and may facilitate standardized treatment algorithms based on objective thrombus burden measurements rather than subjective visual assessments.

Rapid and accurate detection of pulmonary arterial emboli represents a fundamental objective in the PE care pathway, as timely diagnosis directly impacts patient outcomes and survival rates (Zsarnoczay et al., 2025). Beyond simple detection, concurrent risk stratification plays a crucial role in facilitating early identification of high-risk PE patients, enabling prompt initiation of appropriate therapeutic interventions that may significantly reduce mortality rates (Dewailly et al., 2010). The AI software utilized in our investigation incorporates automated assessment capabilities for the pulmonary artery obstruction index (PAOI), which serves as an important quantitative measure of clot burden and has been correlated with clinical severity and prognosis. This automated quantification feature represents a significant advantage over traditional visual assessment methods, as it provides objective, reproducible measurements that can inform clinical decision-making and treatment planning.

Despite the promising clinical utility demonstrated by AI systems in PE detection, our analysis revealed that the software continues to generate both false-negative and false-positive results in real-world clinical practice, highlighting areas where further technological refinement is needed. Our detailed analysis of AI diagnostic errors identified several specific patterns and contributing factors that warrant attention: (1) The predominant cause of AI false-negative results was the failure to detect linear, wall-adherent thrombi located at segmental and subsegmental vessel bifurcations. These adherent clots often present characteristic imaging features known as “saddle” or “acute-angled” signs, which typically indicate the presence of fresh thrombus formation and are particularly important for establishing a diagnosis of acute PE (Xi et al., 2025; Thompson et al., 2024). Additionally, pulmonary consolidation secondary to pneumonia and local imaging artifacts that compromise thrombus visualization contributed to AI detection failures. (2) Common causes of AI false-positive results included early contrast enhancement phases and inadequate filling of pulmonary veins during the imaging acquisition, leading to misinterpretation of normal vascular structures as pathological findings (Uchiyama et al., 2025). Other significant contributors to false-positive results included hilar lymphadenopathy causing compression and narrowing of adjacent pulmonary arteries, tumor invasion of vessel walls creating filling defects that mimic emboli, and heterogeneous contrast mixing patterns that can simulate thromboembolic disease. In comparison, radiologist diagnostic errors showed different patterns, with the most frequent cause of missed PE being failure to detect small clots in subsegmental or more distal pulmonary arterial branches, while the predominant cause of false-positive interpretations was misinterpretation of heterogeneous contrast mixing as pathological findings. This analysis underscores the significant value that AI can provide in the diagnostic workflow and emphasizes the critical importance of fostering close collaboration between radiologists and software developers to continuously optimize AI algorithms and improve overall system performance.

Although our multicenter design involved three hospitals employing predominantly 256-slice CT scanners and one center using 128-slice CT, all within Xinjiang, China, this regional and scanner uniformity may limit the generalizability of the results. Local imaging protocols, population characteristics, and hardware configurations can influence both image quality and AI detection performance. Therefore, future studies extending external validations to institutions across different geographic regions and diverse CT scanner models will be essential to establish broader applicability of the AI system.

Some important limitations of our study warrant acknowledgment and consideration. First, as a retrospective analysis, it is inherently constrained by a limited sample size and a restricted number of image readers. To address this, future work will involve multicenter collaboration to conduct prospective, multicenter studies with larger patient cohorts and multiple independent readers, thereby enabling more robust evaluation of the real-world clinical utility and diagnostic accuracy of the AI system. Second, the PAOI and PTBS calculated in this study has not yet been integrated into a validated risk prediction model for patients with high-risk (massive) pulmonary embolism. The next phase of our research will focus on developing an intelligent, CTPA-based risk stratification model that incorporates clinical severity criteria alongside AI-derived imaging biomarkers. Such a model aims to refine clinical decision-making pathways and ultimately reduce mortality in patients with severe pulmonary embolism.

In conclusion, this comprehensive real-world validation study provides robust evidence supporting the clinical utility of an AI-based system for PE detection using CTPA imaging. Our findings demonstrate that AI technology can rapidly and accurately identify pulmonary arterial emboli while serving as a valuable assistive tool to help radiologists improve their diagnostic efficiency and accuracy. The synergistic combination of artificial intelligence capabilities with human radiological expertise represents a promising approach to enhancing PE diagnosis, potentially leading to improved patient outcomes, reduced diagnostic errors, and more efficient healthcare delivery. As AI technology continues to evolve and mature, ongoing research and development efforts will be essential to further optimize these systems and maximize their clinical impact in the management of patients with suspected pulmonary embolism.

## Data Availability

The original contributions presented in the study are included in the article/Supplementary Material, and further inquiries can be directed to the corresponding authors.
