# Supplementary material for: External validation of a commercial AI system for pulmonary embolism detection on chest CTPA: a multicenter study
Source: Front Mol Biosci. 2026 Mar 11;13:1774152. doi: 10.3389/fmolb.2026.1774152 (PMC13012949; doi:10.3389/fmolb.2026.1774152)
Supplement: Supplementary file 1 [file Supplementaryfile1.docx]

**Algorithm Implementation of the uAI DiscoverPE System**

This section comprehensively elaborates on the core technical framework and key processing steps of the commercial AI system used in our study, with the methodological foundation primarily derived from the state-of-the-art PE detection algorithm described in the attached reference^1^. The AI algorithm for pulmonary embolism (PE) detection and quantitative assessment on CTPA images is built on an improved 3D convolutional neural network VB-Net integrated with Transformer architecture, where VB-Net adopts a bottleneck structure to replace the 5×5×5 convolution in the original V-Net for reducing model parameters and accelerating convergence, and the Transformer module compensates for the lack of positional information capture by convolutional networks via positional encoding and self-attention mechanism. The algorithm implements a two-stage segmentation strategy for pulmonary artery (PA) and thrombus identification: the first-stage network performs rough segmentation of the entire pulmonary vascular network from raw CTPA images to extract the PA centerline skeleton and learn the topological structure of pulmonary vessels, while the second-stage network fuses the first-stage output with original images to conduct fine segmentation of PA and thrombus, and a dedicated classifier is applied to eliminate false-positive thrombus regions and improve segmentation accuracy. After segmentation, a binary classification network is used to distinguish the degree of PA obstruction by thrombi (complete occlusion when the thrombus occupies the entire cross-sectional area of PA branches, partial occlusion otherwise), and a PA grading model combining CNN encoder and stacked Transformer is further employed to grade the pulmonary vascular tree into five levels (PA trunk, main PA, lobar, segmental, and subsegmental) by sampling fixed-size blocks along the PA centerline and extracting fused apparent and positional features of image patches. Based on the Qanadli scoring system, the algorithm automatically calculates the semi-quantitative clot burden score (CBS) using the formula:

$$CBS=\sum(n\times d)\div40\times100\%$$

(n refers to the number of segment-level PAs involved in the thrombus, d is 2 for complete occlusion and 1 for partial occlusion) and the quantitative clot burden volume (CBV) by summing the volume of all segmented thrombi, and it also enables automatic reconstruction of the four-chamber heart view from CTPA images to measure the right ventricular diameter/left ventricular diameter (RVd/LVd) ratio for evaluating right ventricular function. The entire model was trained and validated on a multicenter CTPA dataset (2,424 cases in total, split into training, tuning and testing sets at an 8:1:1 ratio) with strict professional annotation (using 3D Slicer/ITK-SNAP, labeling PA and thrombi >1 mm), and optimized with a hybrid loss function and AdamW optimizer to ensure its performance in clinical PE detection, quantitative assessment of thrombus burden and risk stratification. On the independent test set, the algorithm achieved robust performance: for PA segmentation, the Dice Similarity Coefficient (DSC) reached 0.92 ± 0.03 and the Intersection over Union (IoU) was 0.86 ± 0.04; for thrombus segmentation, the DSC was 0.78 ± 0.06 (0.89 ± 0.03 for central PE, 0.71 ± 0.07 for subsegmental PE) with a sensitivity of 0.85 and specificity of 0.97. For binary PE classification, the area under the ROC curve (AUC) was 0.96, with an overall accuracy of 0.94. Quantitatively, the AI-derived CBV showed a strong positive correlation with manual volumetric measurements (Pearson’s r = 0.91, p < 0.001), and the RVd/LVd ratio measurement achieved an intraclass correlation coefficient (ICC) of 0.93 with expert manual measurements, confirming its reliability for clinical risk stratification.

**References:**

1. Qiao Y, Gao Y, Chen Y, Ye X, Yan C, Zeng M. Quantitative assessment and risk stratification of random acute pulmonary embolism cases using a deep learning model based on computed tomography pulmonary angiography images. *Quant Imaging Med Surg*. Mar 3 2025;15(3):1950-1962. doi:10.21037/qims-24-1412

**Typical examples of misclassified cases**


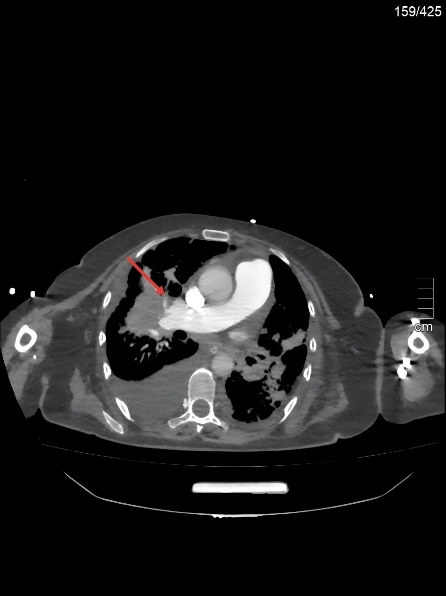

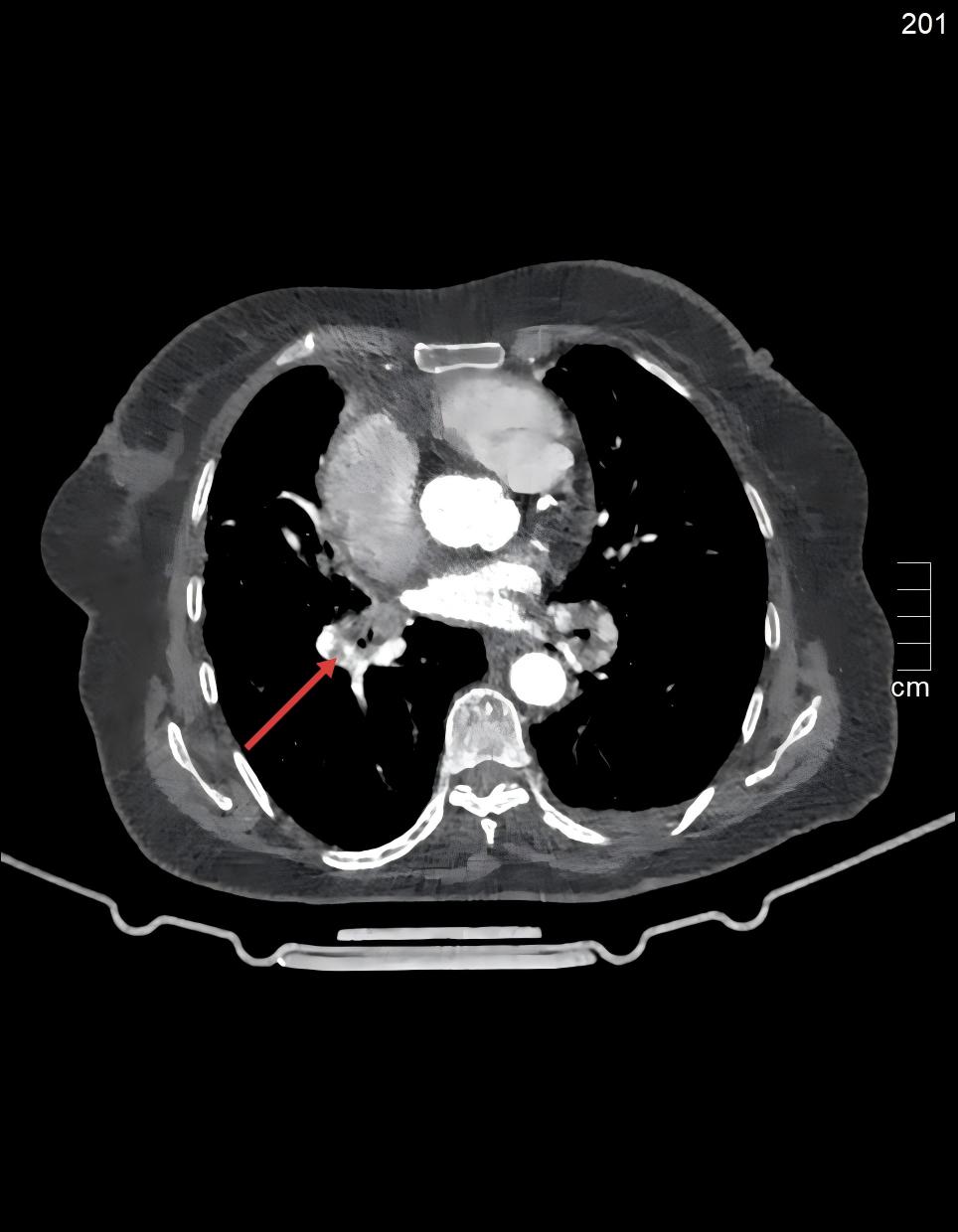

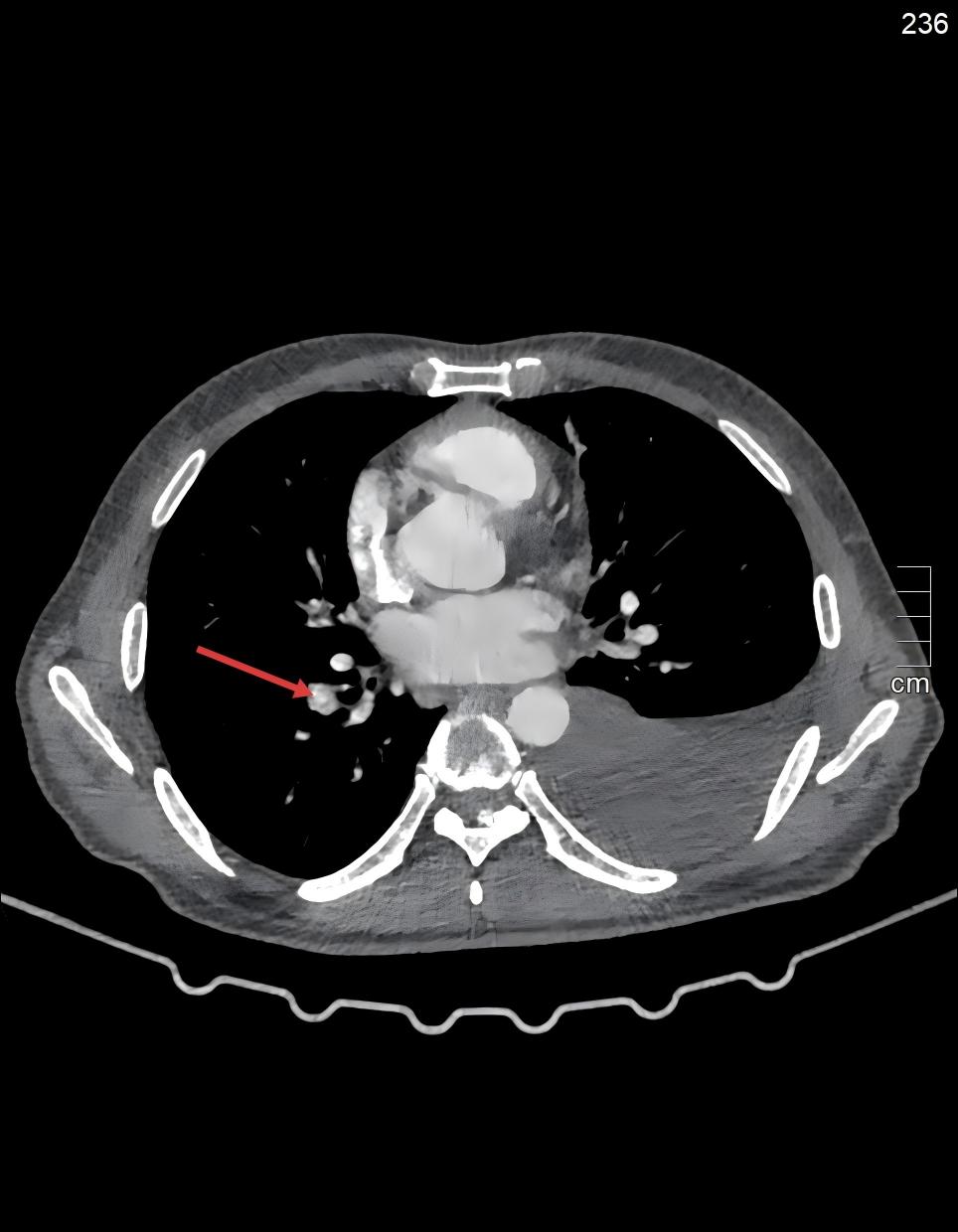


A

B

C

**A: Pulmonary Lesions Causing Artifact-Related Misclassification as Pulmonary Embolism**

Certain pulmonary lesions generated image artifacts or irregularities that interfered with accurate assessment. These artifacts created false signal voids or shadowing that mimicked the appearance of thromboembolic clots within the lung vasculature, resulting in incorrect classification by the algorithm.

**B: Severe Respiratory Motion Artifacts Leading to Misdiagnosis of Pulmonary Embolism**

Significant artifacts caused by respiratory motion during imaging introduced blurring and distortion, which reduced image clarity. These motion-induced abnormalities were misinterpreted by the AI as vascular filling defects consistent with emboli, raising the false-positive rate in affected studies.

**C: Uneven Distribution of Contrast Agent Resulting in False Pulmonary Embolism Identification**

Heterogeneous or patchy distribution of contrast material during the scan produced localized areas of hypoattenuation. The AI system mistook these uneven contrast enhancements as intravascular defects, causing incorrect labeling of vascular segments as embolized.
